# Comprehensive analysis of TPX2-related ceRNA network as prognostic biomarkers in lung adenocarcinoma

**DOI:** 10.7150/ijms.49053

**Published:** 2020-09-01

**Authors:** Chen Huo, Meng-Yu Zhang, Rui Li, Xi-Jia Zhou, Ting-Ting Liu, Jian-Ping Li, Xiao Liu, Yi-Qing Qu

**Affiliations:** 1 Department of Pulmonary and Critical Care Medicine, Qilu Hospital, Cheeloo College of Medicine, Shandong University, Jinan 250012, China.; 2 Department of Pulmonary and Critical Care Medicine, Qilu Hospital of Shandong University, Jinan 250012, China.

**Keywords:** Lung adenocarcinoma, TPX2, competing endogenous RNA (ceRNA), prognostic biomarkers

## Abstract

**Background and aim:** Competing endogenous RNA (ceRNA) is believed to play vital roles in tumorigenesis. The goal of this study was to screen prognostic biomarkers in lung adenocarcinoma (LUAD).

**Methods:** Common differentially expressed genes (DEGs) were collected from Gene Expression Omnibus (GEO) databases and The Cancer Genome Atlas databases (TCGA) using GEO2R and “limma” package in R, respectively. Overlapping DEGs were conducted using enrichment of functions and protein-protein interaction (PPI) network to discover significant candidate genes. By using a comprehensive analysis, we constructed an mRNA mediated ceRNA network. Survival rates were used Kaplan-Meier analysis. Statistical analysis was used to further identify the prognosis of studied genes.

**Results:** Integrated analysis of GSE32863 and TCGA databases, a total of 886 overlapping DEGs, including 279 up-regulated and 607 down-regulated genes were identified. Considering the highest term of candidate genes in PPI, we identified TPX2, which was enriched in cell division signaling pathway. Besides, 35 differentially expressed miRNAs (DEmiRNAs) were predicted to target TPX2 and only 7 DEmiRNAs were identified to be prognostic biomarkers in LUAD. Then, 30 differentially expressed lncRNAs (DElncRNAs) were predicted to bind these 7 DEmiRNAs. Finally, we found that 7 DElncRNAs were correlated with the overall survival (all *p* <0.05). Furthermore, we identified elevated TPX2 was strongly correlated with the worse survival rate among 458 samples. Univariate and multivariate cox analysis showed TPX2 may act as an independent factor for prognosis in LUAD (*p* <0.05). Then pathway enrichment results suggested that TPX2 may facilitate tumorigenesis by participating in several cancer-related signaling pathways in LUAD, especially in Notch signal pathway.

**Conclusions:** TPX2-related lncRNAs and miRNAs are related to the survival of LUAD. 7 lncRNAs, 7 miRNAs and TPX2 may serve as prognostic biomarkers in LUAD.

## Introduction

Lung cancer is the main cause of cancer-related death, and lung adenocarcinoma (LUAD) is one of histologic type, which account for about half of lung cancer 1-4. Despite the advances in new targeted therapies, chemotherapy, radiotherapy as well as surgical methods, the 5-year survival rate of LUAD is 20% 5-7, which illustrate the urgency to find biomarkers for prognosis of LUAD. So it is vital to identify potential molecular mechanisms and significant prognostic biomarkers in LUAD.

Long non-coding RNAs (lncRNAs) with longer than 200 bp could interact with mRNAs, miRNAs and circular RNAs, participating in complicated regulation network of gene expression. LncRNAs' abnormal expressions have linked with various cancers, for instance, prostate cancer, colorectal cancer and pancreatic cancer 8-12. MiRNAs are classes of short RNA molecules with 19 to 25 nucleotides, which regarded as one of the regulators of gene expression and silenced target genes in post-transcriptional levels. Growing evidences suggested that miRNAs participate in biological process, for instance, DNA damage response, cell proliferation, cell apoptosis 13-15. With further understanding of biological process, a new regulatory mechanism named competing endogenous RNA (ceRNA) was proposed, which provides a better insight into tumorigenesis 16. CeRNA can bind to miRNA by miRNA response elements (MREs) to induce gene silencing. More and more researches demonstrated that ceRNA network plays crucial roles in numerous cancers 17-18.

Targeting protein for Xenopus kinesin-like protein 2 (TPX2) is a microtubule-associated protein, which was involved in mitotic spindle formation 19-20. Growing evidence has proved that increased TPX2 contribute to the poor prognosis by triggering spindle dysfunctions, subsequent chromosomal instability or abnormal DNA damage response in tumor development 21. TPX2 overexpression is positively correlated with lymphatic metastasis, tumor grade and stage, negatively with the survival rate 22-24. However, there are no systematic studies to investigate the prognostic value of TPX2 in LUAD. This study aims to construct a critical TPX2-related ceRNA network in LUAD.

## Material and Methods

### Data collection and significant differentially expressed RNAs screening

The gene expression profile of GSE32863 based on the GPL6884 platform, was selected from Gene Expression Omnibus (GEO) (http://www.ncbi.nlm.nih.gov/geo/) 25. GSE32863 included 58 pairs of LUAD and normal lung tissues, normalized microarray data was reanalyzed using the GEO2R online tools (https://www.ncbi.nlm.nih.gov/geo/geo2r/) 26. The Cancer Genome Atlas (TCGA) (http://cancergenome.nih.gov/) contains expression profiles and prognostic information, so we gathered RNA-sequencing data of LUAD as well. Then “limma” package in R was performed to identify differentially expressed lncRNAs (DElncRNAs), differentially expressed miRNAs (DEmiRNAs) and differentially expressed genes (DEGs). Volcano plot was generated using the “ggplot2” R package, and the “Venn Diagram” package was performed to overlap DEGs. Down-regulated gene was defined as logFC<0, on the contrary, up-regulated was defined as log FC >0. The significant differentially expressed RNAs were defined as p< 0.01 and |logFC| > 1, statistically.

### The functional enrichment and pathway analysis

Gene ontology (GO) analysis is a common method to identify functional enrichment of gene data. And the function and pathway analysis were constructed through Database for Annotation, Visualization and Integrated Discovery (DAVID) (https://david.ncifcrf.gov/). Then functional enrichment of candidate genes were visualized using “GOplot” R package. As for signaling pathway analysis, “cluster profile” R package was used to discover significant pathways. *p* < 0.05 was used to significant terms statistically. To identify the potential function of TPX2, Gene set enrichment analysis (GSEA) (http://software.broadinstitute.org/gsea/index.jsp) was conducted between the low-expression and high-expression groups 27. And *p* < 0.05 was regarded as statistically significant.

### Candidate gene selection

Search Tool for the Retrieval of Interacting Genes (STRING) (http://string-db.org/) was used to obtain the predicted interactions for overlapping DEGs with confidence score >0.4 28, protein-protein interaction (PPI) network of overlapping DEGs was visualized by Cytoscape 29. The CytoHubba plugin gave us a novel insight into the importance of networks by ranking nodes 30, according to which we found hub genes. The Molecular Complex Detection (MCODE) plugin was performed to select linked genes with degree cutoff = 2 and k-core = 2.

### Construction of a TPX2-related ceRNA network

LncRNA and mRNA have the same miRNA response elements (MREs) by competitively binding to miRNA to regulate each other's expression. In this study, a ceRNA network of lncRNAs, miRNAs, and TPX2 was constructed to explore their functions. The relationship between TPX2 and target miRNAs was analyzed by using three databases miRTarBase (http://mirtarbase.mbc.nctu.edu.tw/), TargetScan (http://www.targetscan.org/) and StarBase (http://starbase.sysu.edu.cn/). The unions of the prediction results of three databases were used to perform further analysis. LncRNAs targeted by the miRNAs were retrieved from the StarBase.

### Statistical analysis

Statistical analysis was performed using SPSS and R 3.6.2 software. The differences of TPX2 expression levels between the LUAD and normal lung groups were determined by Student's *t*-test. The Chi-square test was used to analyze the associations between TPX2 expression and clinicopathological factors of LUAD patients. The survival R packages were performed for survival analysis of TPX2 and survival curve was displayed by Kaplan-Meier plotter and examined by log-rank test. Univariate and multivariate Cox analysis were used to evaluate the prognostic value between TPX2 and other clinicopathological fators. Lung cancer explorer (LCE) database (http://lce.biohpc.swmed.edu/lungcancer/index.php#page-top) includes gene expression and clinical data from more than 6,700 lung cancer patients, which provides forest plots to summarize tumor-normal standardized mean difference and hazard ratios 31. The *p* < 0.05 was considered to be statistically significant.

## Results

### Differentially expressed RNAs in LUAD

**Figure 1:**
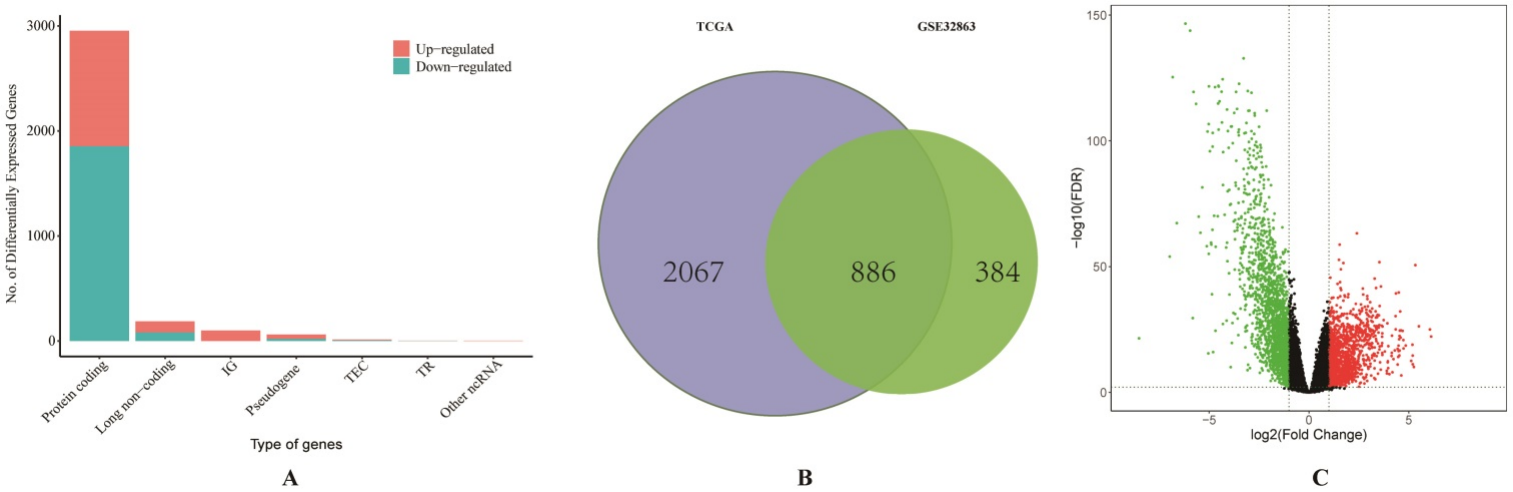
** Comprehensive analysis of differentially expressed RNAs. (A)** The bar graph of the proportion of all differentially expressed RNAs from TCGA database.** (B)** Identification of differential expressed genes from TCGA and GSE32863.** (C)** The volcano plot of differentially expressed genes from TCGA database.

We firstly obtained the gene expression profile of 58 pairs of LUAD tissues and their matched normal lung tissues from GSE32863. A total of 1270 DEGs were identified by using GEO2R. Then the RNA-sequencing data and clinical data of LUAD were downloaded from TCGA. Based on the differentially expressed RNAs analysis by limma R package, a total of 186 DElncRNAs, 150 DEmiRNAs and 2953 DEGs were identified, and the proportion of all differentially expressed RNAs from TCGA was visualized (**Figure 1A**). As it shown in Figure 1C, the result of volcano plot displayed the distribution of DEGs in the dimensions of -log (FDR) and logFC. On the basis of *p* <0.01 and |Log2(FC)|>1, a total of 886 overlapping DEGs, including 279 up-regulated and 607 down-regulated genes, were screened from two studies (**Figure 1B**).

### Enrichment analysis of DEGs in LUAD

**Figure 2:**
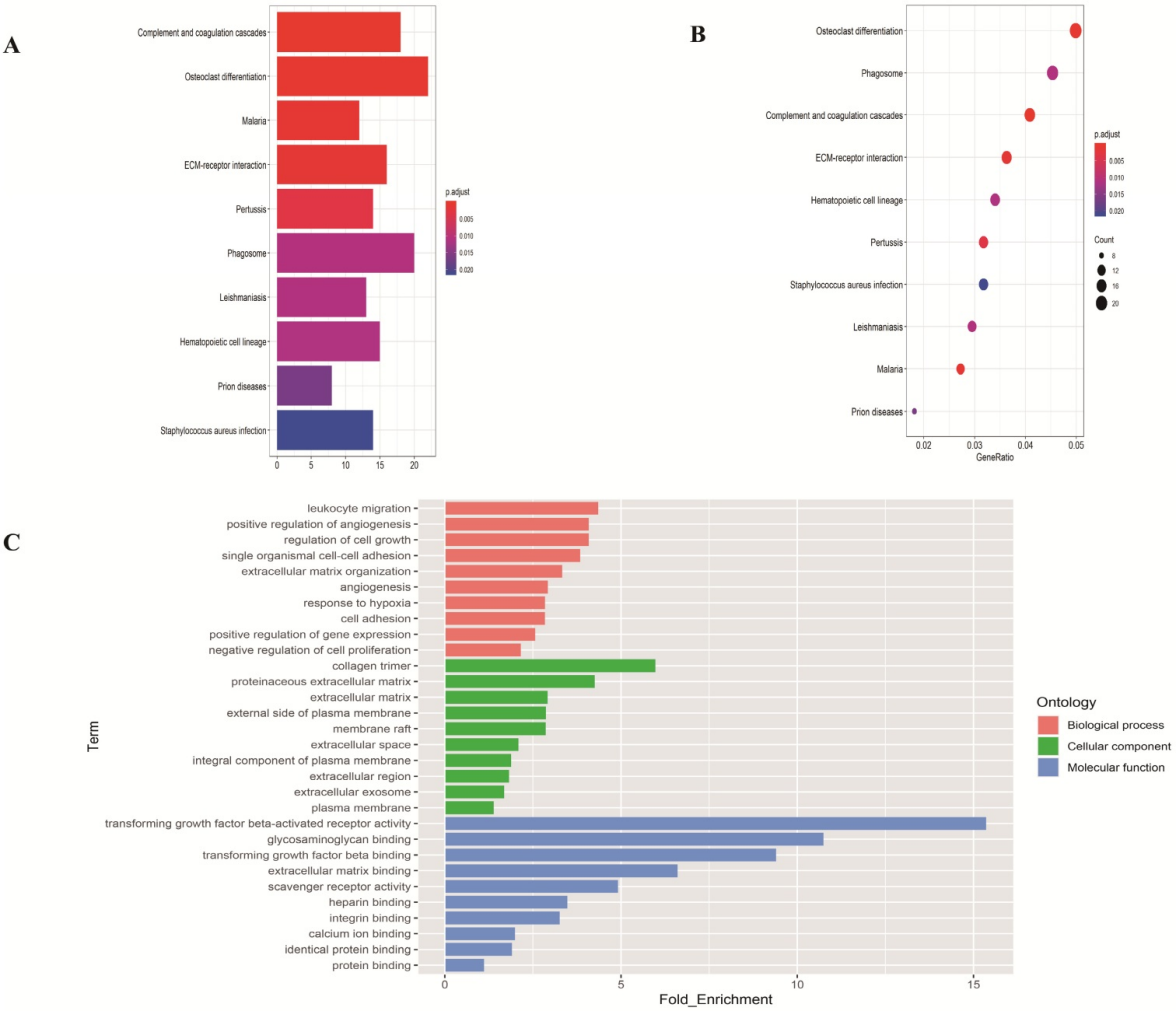
** Functional enrichment analysis of overlapping differentially expressed genes in LUAD. (A-B)** The top ten pathways of overlapping differentially expressed genes in barplot and dotplot. **(C)** Gene Ontology terms of overlapping differentially expressed genes.

To investigate the function of the DEGs, GO and KEGG analysis were performed using the web-based tool DAVID. Enriched terms with *p* <0.05 were displayed (**Figure 2**). The genes were mainly enriched in leukocyte migration and positive regulation of angiogenesis in the biological process; collagen trimer and proteinaceous extracellular matrix in the cellular component; transforming growth factor beta-activated receptor activity and glycosaminoglycan binding in the molecular function.

### Construction of the PPI network and screening of modules and hub genes

**Figure 3:**
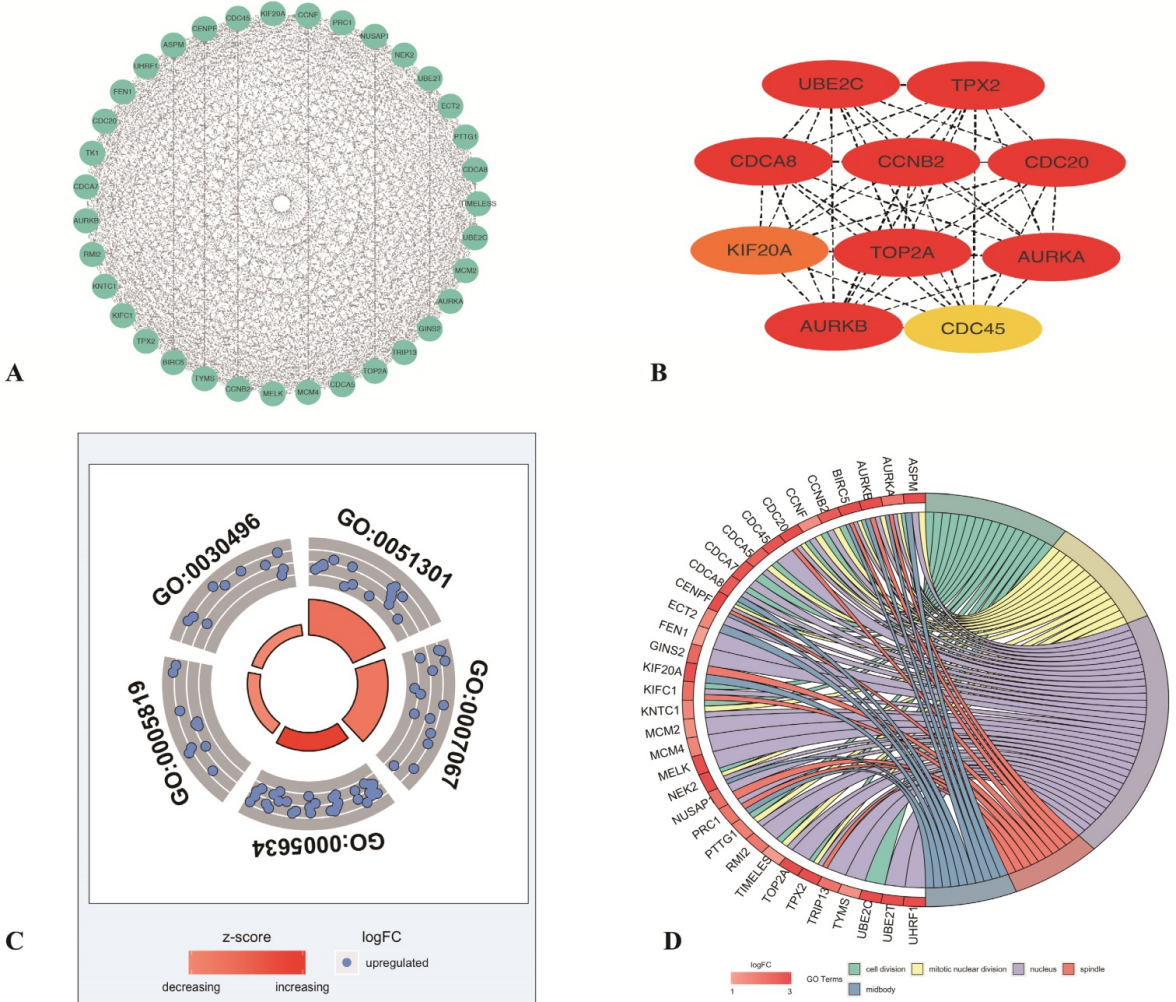
**Screening the key genes and further GO analysis from PPI network. (A-B)** Hub genes selected using MCODE and CytoHubba. **(C)** The outer circle represents the expression (logFC) of 35-differentially expressed genes in each enriched GO (gene ontology) terms. The inner circle indicates the significance of GO terms (log10-adjusted p values). Red points indicate upregulated gene and blue points indicate downregulated gene. **(D)** The circle indicates the correlation between 35 differentially expressed genes and their gene ontology terms.

To identify the most important proteins and biological modules which may be involved in the progression of LUAD the PPI network was generated by overlapping genes using STRING and the interactors of top 10 hub genes were visualized using Cytoscape software. Subsequently, to explore the significance of these DEGs, the top 1 module, which displayed the most densely connected region, was selected (**Figure 3A**). The result showed the top 10 hub nodes (TPX2, TOP2A, AURKB, CCNB2, CDC20, CDCA8, AURKA, UBE2C, KIF20A, CDC45) with the highest degrees in LUAD (**Figure 3B**). And to explore the possible function of these 35 selected DEGs, the functional annotation of 35 genes (PTTG1, TPX2, ASPM, NUSAP1, TOP2A, TYMS, TK1, CDCA7, AURKB, CCNB2, TRIP13, CDC20, NEK2, UHRF1, CDCA8, FEN1, MCM2, MCM4, KNTC1, MELK, BIRC5, CENPF, KIFC1, AURKA, CCNF, TIMELESS, UBE2C, PRC1, UBE2T, KIF20A, CDC45, GINS2, CDCA5, RMI2, ECT2) made by M-code showed that 5 GO terms were statistically significant (*p* <0.05). The result showed the interaction between 35 DEmRNAs and their associated GO terms, in which the highest GO term was cell division (**Figure 3C-D**), and 16 DEGs (KIFC1, CDCA8, CCNB2, CDCA7, TIMELESS, NEK2, CCNF, TPX2, KNTC1, CENPF, BIRC5, AURKA, CDC20, PTTG1, UBE2C, CDCA5) were contained in this term (**Table 1**). Above all, top 10 hub genes, which were contained in these 16 genes, indicated that these proteins may serve crucial roles in the whole protein interaction network. Then in consideration of the enrichment results of highest GO term, TPX2 was selected for further analysis.

### Construction of TPX2-related ceRNA network

**Figure 4:**
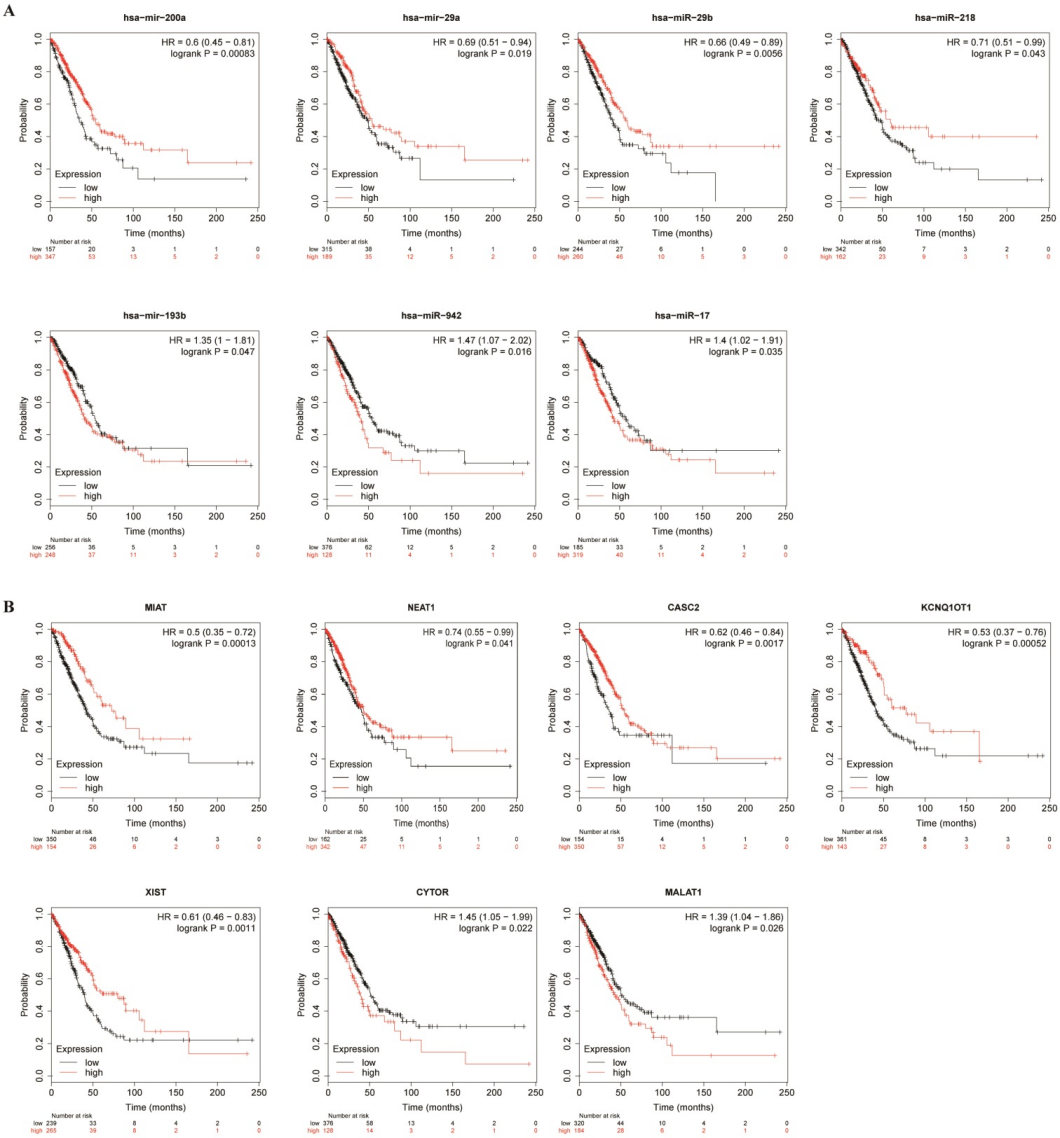
** Kaplan-Meier curve analysis of TPX2-related DElncRNAs and DEmiRNAs in LUAD. (A)** Prognostic values of DEmiRNAs in LUAD.** (B)** Prognostic values of DElncRNAs in LUAD.

**Figure 5:**
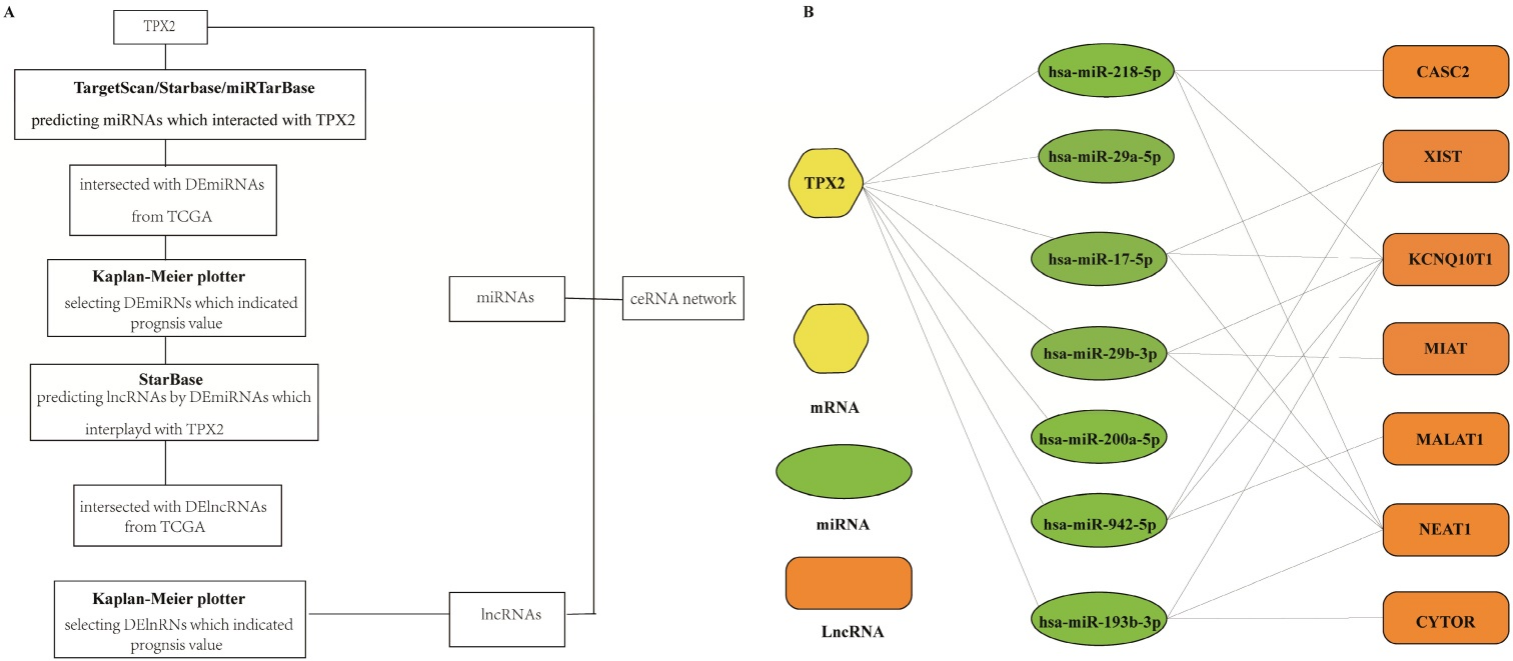
** (A)** A flow diagram of TPX2-related ceRNA network.** (B)** Construction of lncRNA-miRNA-TPX2 network in LUAD.

To better predict the upstream regulation mechanism of TPX2, we constructed a ceRNA network. At the beginning, the miRNAs targeted by TPX2 were predicted via StarBase, miRTarBase and TargetScan. We identified a total of 35 DEmiRNAs from TCGA, and we further assessed prognostic values of these 35 predicted DEmiRNAs using Kaplan-Meier plotter database. The results showed that 7 of 35 DEmiRNAs (miR-942-5p, miR-193b-3p, miR-29b-3p, miR-17-5p, miR-218-5p, miR-29a-5p and miR-200a-5p) were significantly associated with overall survival (all *p* <0.05) (**Figure 4A**). Subsequently, the lncRNAs targeted by 7 DEmiRNAs were predicted via StarBase. A total of 30 DElncRNAs were predicted, and only 7 of 30 DElncRNAs (KCNQ1OT1, XIST, MALAT1, CYTOR, NEAT1, CASC2, MIAT) have prognostic values (all *p* <0.05) (**Figure 4B**). Ultimately, we constructed the TPX2-related ceRNA network of LUAD, including 7 DElncRNAs, 7 DEmiRNAs as well as TPX2, which functioned as prognostic biomarkers for LUAD patients (**Figure 5A-B**).

### TPX2 expression and correlation with clinical characteristics

**Figure 6:**
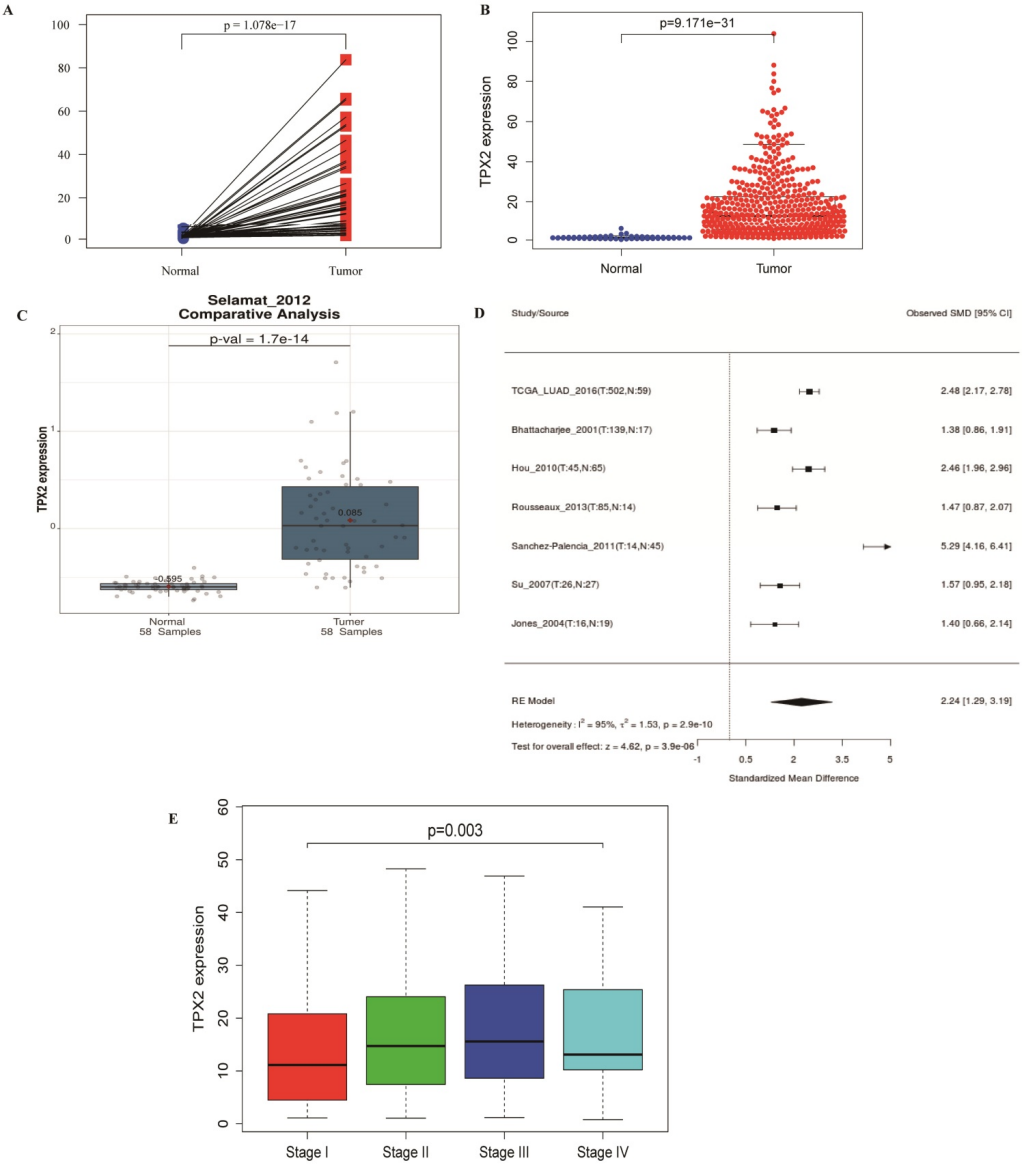
** The relationship between TPX2 expression and clinical characteristics in LUAD. (A-C)** Expression of TPX2 in LUAD patients based on TCGA and GSE32863. **(D)** Meta-analysis for the expression of TPX2 in LUAD and normal lung tissues. **(E)** The relation between TPX2 expression and tumor stage in LUAD patients.

486 LUAD and normal lung samples from the TCGA database were enrolled, and a simple summary of the data was showed in **Table 2.** The expression of TPX2 in LUAD tissues was significantly up-regulated compared with normal tissues (*p* <0.001), which is consistent with GEO database (**Figure 6A-C**). The expression of TPX2 in the 7 independent datasets was pooled in a forest plot (**Figure 6D**). The SMD was 1.40 (95% CI: 1.29-3.19; I^2^ =95%; *p* = 2.0e-10), which suggested that the occurrence of LUAD is related to expression levels of TPX2. To determine whether TPX2 expression level was related to LUAD progression, expression level of TPX2 from 458 patients was analyzed. Clinical characteristics and their relations with TPX2 were performed, and the results showed TPX2 expression level was significantly associated with gender (*p*=0.005), stage (*p*=0.001), pathological T stage (*p*=0.018) and pathological N stage (*p*=0.004). However, no significant difference was found in age (*p*=0.133) and pathological M stage (*p*=0.703) (**Table 3**). Figure 6E showed that the expression level of TPX2 displayed strong correlation with the tumor stage in patients with LUAD (*p* <0.05).

### Prognostic significance of TPX2 in LUAD

**Figure 7:**
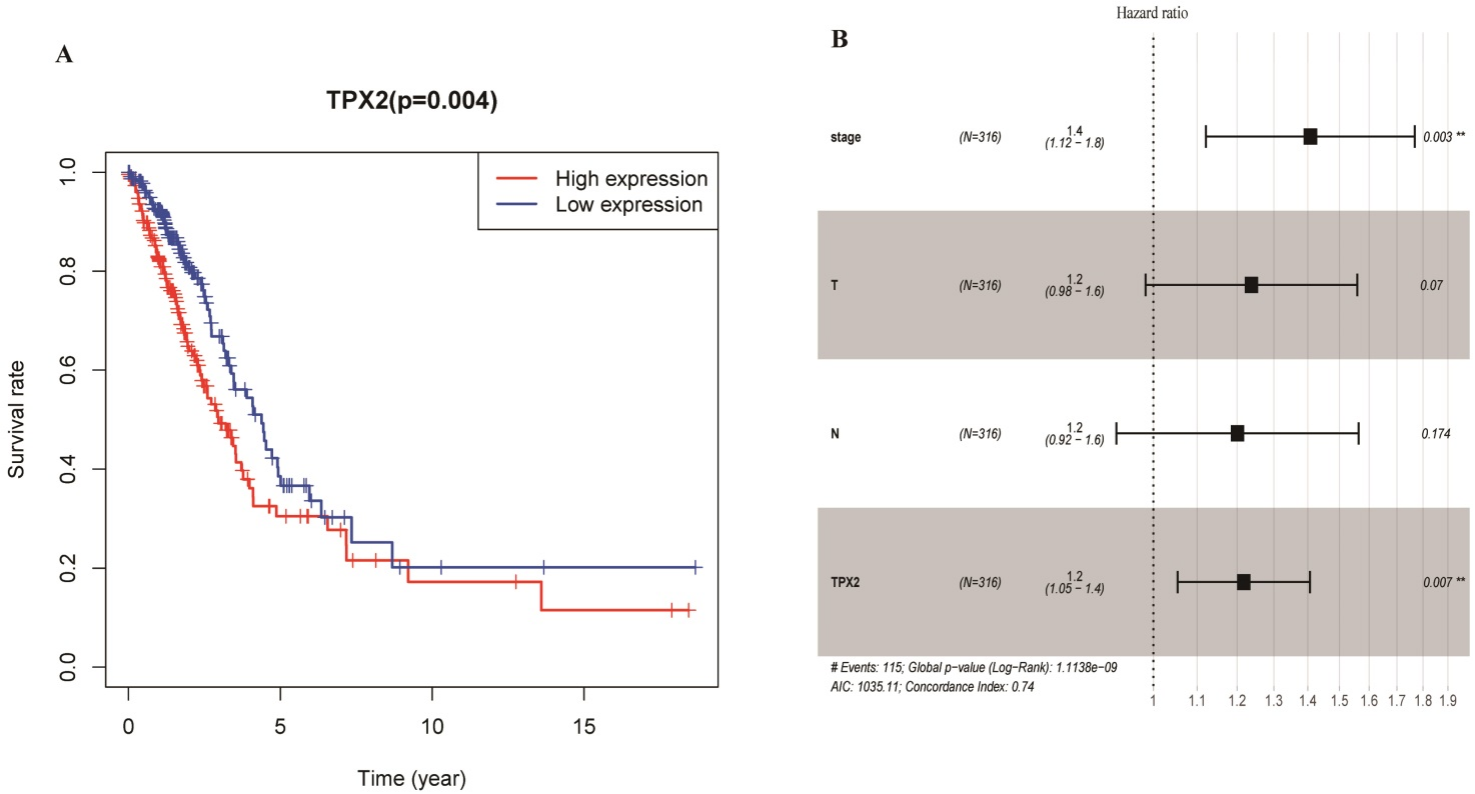
** Expression of TPX2 in LUAD was correlated with prognosis. (A)** Overall survival analysis of LUAD patients with different TPX2 expression.** (B)** Forest plot of multivariate cox analysis in LUAD.

To elucidate the prognostic values of TPX2, we assessed the correlation between TPX2 expression level and overall survival rate in LUAD. On the basis of the median risk scores, a total of 458 patients were divided into a high risk group and a low risk group. The results showed that high expression of TPX2 was associated with worse overall survival for LUAD patients (*p*=0.004) (**Figure 7A**). Moreover, we used univariate and multivariate analysis to distinguish the risk factors correlated with the prognosis of LUAD patients by using Cox regression model. Univariate independent prognostic analysis showed that clinical stage, pathological T stage, pathological N stage and expression levels of TPX2 were statistically significant with overall survival (*p* <0.05). Multivariate independent prognostic analysis showed that clinical stage and the TPX2 expression can act as independent prognostic factors (*p* <0.05) (**Figure 7B, Table 4**).

### Gene Set Enrichment Analysis of TPX2

**Figure 8:**
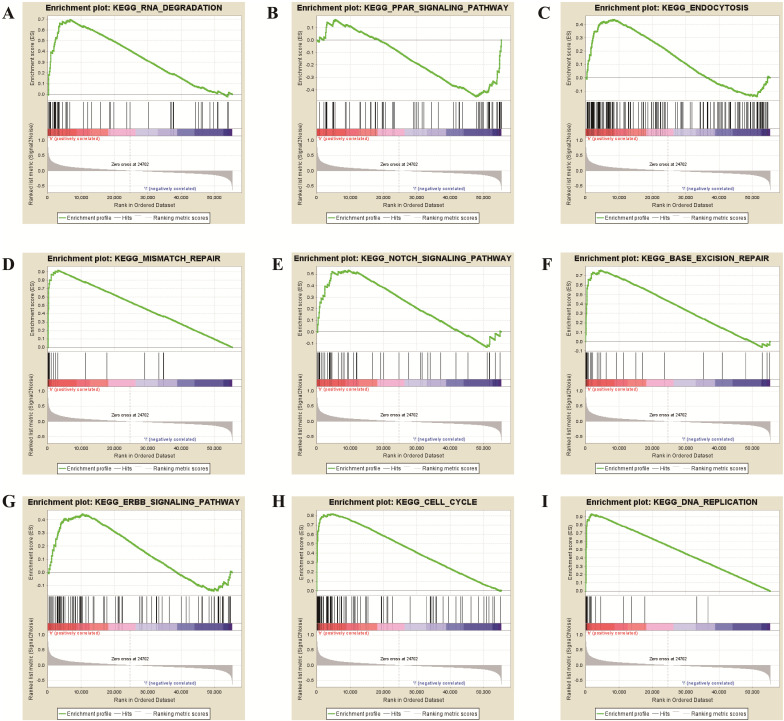
** GSEA revealed that TPX2 was enriched in pathways of (A)** RNA Degradation, **(B)** PPAR signaling pathway, **(C)** Endocytosis, **(D)** Mismatch Repair, **(E)** Notch signaling pathway, **(F)** Base Excision Repair, **(G)** ERBB signaling pathway, **(H)** Cell cycle, **(I)** DNA Replication.

To further verify the potential function of TPX2 gene, GSEA was used to obtain the biological process. Ultimately, TPX2 was associated with cancer-related signaling pathways including “cell cycle”, “RNA degradation”, “mismatch repair”, “DNA replication”, “base excision repair”, “endocytosis”, “Notch signaling pathway” “PPAR signaling pathway” and “ERBB signaling pathway” (all *p* <0.05) (**Figure 8, Table 5**). Enrichment plots of GSEA demonstrated that the gene signatures of “Notch signaling pathway” (NES = 1.76) with higher TPX2 expression were more active than lower TPX2 expression (*p* <0.05, FDR <0.05). All these results suggested that TPX2 may facilitate tumorigenesis by taking part in several cancer-related signaling pathways in LUAD.

## Discussion

Lung cancer is the chief cause of cancer death (18.4% of the 9.6 million cancer death) 32, and LUAD is the most common subtype of lung cancer. Despite advances in the diagnosis and treatment, the overall survival rates remain poor in LUAD 33. Recent studies have identified novel molecular biomarkers may provide a prognosis value in combination with patients' clinical parameters 34-35, and in which non-coding RNAs (ncRNAs) play vital roles in cancer initiation and progression 36. For example, LncRNA DGCR5 promotes LUAD progression 37, miR-1323 promotes cell migration in LUAD 38. With the increasing understanding of ceRNA, more and more people have studied the mechanism of ceRNAs in cancer. Yang et al. indicated that lncRNA LCAT1 regulates RAC1 in lung cancer 39. And lncRNA WDFY3-AS2 promotes LUAD progression via targeting miR-491-5p/ZNF703 axis 40. In this present study, we firstly constructed a new mRNA-miRNA-lncRNA regulatory network using RNA sequencing data and microchip data, in which each RNA in the network has important implications for the prognosis of LUAD.

A total of 886 significant DEGs were identified, which mainly enriched in cell growth, cell adhesion and metabolic pathways. Considering the results of functional enrichment analysis, these significant DEGs may regulate growth of LUAD. We obtained 35 core genes from PPI network and these genes were re-analyzed for functional enrichment, in which cell division were chosen for further study because it is the most significant GO terms (*p*=1.44E-13). Finally, 16 DEGs were significantly enriched in the cell division which is closely related to tumorigenesis. Considering the highest term of candidate genes in PPI, we identified TPX2 for further study. Besides, we discovered that most of these genes have been well studied except TPX2. For example, AURKA promotes tumor proliferation and was associated with worse prognosis in bladder cancer and gastrointestinal cancer 41-42. Increased AURKB have a poor prognosis in NSCLC 43. CDCA8 participated in lung carcinogenesis 44. KIF20A promoted cell proliferation and inhibit apoptosis in LUAD 45. And UBE2C was found to enhance lung cancer growth 46.

Subsequently, we predicted 30 DElncRNAs and 35 DEmiRNAs, in which 7 DEmiRNAs and 7 DElncRNAs functioned as prognostic biomarkers in LUAD. High expression levels of 4 DEmiRNAs (miR-218-5p, miR-29b-3p, miR-200a-5p, miR-29a-5p) have a better prognosis and high expression levels of 3 DEmiRNAs (miR-17-5p, miR-942-5p, miR-193b-3p) were associated with worse prognosis in LUAD. In the past studies, miR-218-5p inhibited proliferation and migration of NSCLC 47. LncRNA H19 was found to promote epithelial-mesenchymal transition of LUAD by targeting miR-29b-3p and modifying STAT3 48. Wang A et al. proved that miR-29a-5p regulated TETs in colorectal cancer 49. MiRNA-17-5p inhibited triggers apoptosis in NSCLC 50. Then we predicted 30 DElncRNAs of 7 DEmiRNAs, and only 7 DElncRNAs were significantly associated with overall survival. Low expression levels of 2 DElncRNAs (MALAT1 and CYTOR) increased survival times. High expression levels of 5 DElncRNAs (KCNQ1OT1, XIST, NEAT1, CASC2, MIAT) have a better prognosis. In previous studies, KCNQ1OT1 indicated a better prognosis in lung cancer 51. MALAT1 promoted the proliferation and invasion of NSCLC 52. Finally, a prognosis-associated TPX2-DEmiRNA-DElncRNA network in LUAD was established.

Then our findings revealed that overexpressed TPX2 is correlated with poor prognosis in LUAD. Moreover, univariate and multivariate cox model proved that TPX2 expression can act as an independent factor for prognisis. For example, TPX2 is an independent prognostic factor in esophageal cancer 53-54, digestive system cancer 55 and clear cell renal cell carcinoma 56. Furthermore, our analysis indicated TPX2 was mostly concentrated in tumor-related pathways. Among results of pathway, we ultimately focused on the Notch pathway, which is connected with cell proliferation, differentiation and survival. And Notch pathway played a crucial role in the development of multiple cancers 57-58. Previous study showed that activation of the Notch pathway can lead to a malignant phenotype of lung cancer 59. It has been noted that Notch1 signaling was reported to be activated in NSCLC 60. Another study proved that Notch2 has a tumor suppressive effect in NSCLC 61. Hassan et al. presented that Notch3 behaves as a tumor promoter pathway in NSCLC 62.

In conclusion, we constructed a novel TPX2-related ceRNA regulatory network by comprehensive bioinformatics analysis, in which all RNAs could be prognostic biomarkers of LUAD. However, these biomarkers need to be further validated by molecular biology experiments.

## Figures and Tables

**Table 1:** The top5 GO terms of 35 differentially expressed genes

Category	ID	Term	Genes	adj_pval
BP	GO:0051301	cell division	KIFC1, CDCA8, CCNB2, CDCA7, TIMELESS, NEK2, CCNF, TPX2, KNTC1, CENPF, BIRC5, AURKA, CDC20, PTTG1, UBE2C, CDCA5	1.44E-13
BP	GO:0007067	mitotic nuclear division	CCNB2, TIMELESS, NEK2, CCNF, KNTC1, TPX2, CENPF, AURKA, BIRC5, CDC20, PTTG1, AURKB, CDCA5, ASPM	1.03E-12
CC	GO:0005634	nucleus	KIFC1, PRC1, NEK2, KNTC1, AURKA, PTTG1, AURKB, TYMS, CDC45, CDCA8, CDCA7, TOP2A, CDCA5, ASPM, FEN1, TRIP13, GINS2, CCNF, TPX2, NUSAP1, CENPF, RMI2, BIRC5, CDC20, MCM2, ECT2, MCM4, UHRF1, CCNB2, TIMELESS, MELK, UBE2T	1.01E-10
CC	GO:0005819	spindle	KIFC1, PRC1, TPX2, NUSAP1, CENPF, AURKA, BIRC5, CDC20, AURKB, KIF20A	9.10E-10
CC	GO:0030496	midbody	CDCA8, PRC1, NEK2, CENPF, AURKA, BIRC5, AURKB, ECT2, ASPM, KIF20A	1.63E-09

**Table 2:** Clinical characteristics of LUAD patients obtained from TCGA database

Clinical characteristics	Variable	Patients, n (%)
Age	>65	240 (49)
	≤65	227 (47)
	unknown	19 (4)
Gender	Male	222 (46)
	Female	264 (54)
Pathology stage	I	262 (54)
	II	112 (23)
	III	79 (16)
	IV	25 (5)
	unknown	8 (2)
Pathology T stage	T1	163 (34)
	T2	260 (53)
	T3	41 (8)
	T4	19 (4)
	unknown	3 (1)
Pathology M stage	M0	333 (69)
	M1	24 (5)
	Mx	129 (26)
Pathology N stage	N0	312 (64)
	N1	90 (19)
	N2	70 (14)
	N3	2 (1)
	Nx	12 (2)

**Table 3:** Association between TPX2 expression levels and clinicopathological characteristics of LUAD patients

Characteristic	n=458	TPX2		
		Low (n=229)	High (n=229)	*p* value
**Age (years)**				0.133
<65	206	95	111	
≥65	252	134	118	
**Gender**				**0.005**
Male	208	89	119	
Female	250	140	110	
**Stage**				**0.001**
I	246	143	103	
II	105	39	66	
III	74	30	44	
IV	25	11	14	
unknown	8	6	2	
**Pathological T stage**				**0.018**
T1	158	96	62	
T2	239	105	134	
T3	39	17	22	
T4	19	9	10	
unknown	3	2	1	
**Pathological N stage**				**0.004**
N0	295	159	136	
N1	84	34	50	
N2+3	67	26	41	
Nx	12	10	2	
**Pathological M stage**				0.703
M0	305	154	151	
M1	24	10	14	
unknown	129	65	64	

**Table 4:** Univariate and multivariate analysis of prognostic factors in LUAD

Variables	Univariate analysis		Multivariate analysis	
	Hazard ratio (95% CI)	*p* value	Hazard ratio (95%CI)	*p* value
Age	1.002 (0.983-1.021)	0.843		
Gender	1.035 (0.717-1.495)	0.852		
Stage	1.654 (1.401-1.951)	<0.01	1.408 (1.121-1.768)	0.003
T	1.632 (1.315-2.024)	<0.01	1.238 (0.983-1.560)	0.070
M	1.757 (0.964-3.203)	0.066		
N	1.790 (1.459-2.196)	<0.01	1.201 (0.922-1.564)	0.174
TPX2	1.010 (1.003-1.017)	0.003	1.218 (1.054-1.4077)	0.007

CI: confidence interval.

**Table 5:** The significant enriched signaling pathways from GSEA results (*p* < 0.05)

NAME	ES	NES	NOM p-val	FDR q-val
CELL CYCLE	0.818	2.690	0.000	0.000
OOCYTE MEIOSIS	0.649	2.499	0.000	0.000
HOMOLOGOUS RECOMBINATION	0.897	2.417	0.000	0.000
UBIQUITIN MEDIATED PROTEOLYSIS	0.591	2.397	0.000	0.000
SPLICEOSOME	0.788	2.390	0.000	0.000
NUCLEOTIDE EXCISION REPAIR	0.765	2.370	0.000	0.000
RNA DEGRADATION	0.696	2.364	0.000	0.000
P53 SIGNALING PATHWAY	0.604	2.315	0.000	0.000
MISMATCH REPAIR	0.917	2.313	0.000	0.000
BASAL TRANSCRIPTION FACTORS	0.714	2.257	0.000	0.001
PYRIMIDINE METABOLISM	0.642	2.254	0.000	0.001
DNA REPLICATION	0.933	2.206	0.000	0.001
PENTOSE PHOSPHATE PATHWAY	0.732	2.145	0.000	0.002
PROTEASOME	0.859	2.129	0.000	0.002
BASE EXCISION REPAIR	0.759	2.099	0.000	0.003
PATHWAYS IN CANCER	0.467	2.034	0.002	0.006
FC GAMMA R MEDIATED PHAGOCYTOSIS	0.501	1.921	0.002	0.015
PROSTATE CANCER	0.478	1.846	0.008	0.024
REGULATION OF ACTIN CYTOSKELETON	0.452	1.829	0.008	0.027
RNA POLYMERASE	0.629	1.810	0.012	0.030
ENDOCYTOSIS	0.437	1.793	0.006	0.032
NOTCH SIGNALING PATHWAY	0.534	1.759	0.014	0.041
GAP JUNCTION	0.455	1.749	0.011	0.042
PROTEIN EXPORT	0.637	1.730	0.031	0.046
ERBB SIGNALING PATHWAY	0.445	1.724	0.014	0.046
PRION DISEASES	0.545	1.722	0.021	0.046
ASTHMA	-0.729	-1.765	0.046	0.120
PPAR SIGNALING PATHWAY	-0.455	-1.642	0.008	0.151

## Abbreviations

DAVID: The Database for Annotation, Visualization and Integrated Discovery
GO: Gene ontology
KEGG: Kyoto Encyclopedia of Genes and Genomes
NSCLC: Non-small-cell lung cancer
LUAD: Lung adenocarcinoma
TPX2: Targeting protein for Xenopus kinesin-like protein 2
FDR: False discovery rate
ceRNA: Competing endogenous RNA
TCGA: The Cancer Genome Atlas Project
GEO: The Gene Expression Omnibus database
LCE: Lung cancer explorer
DElncRNAs: differentially expressed lncRNAs
DEmiRNAs: differentially expressed miRNAs
DEGs: differentially expressed genes
ncRNAs: non-coding RNAs
